# The importance of pre‐ablation atrial septal evaluation for a patient with surgical patch closure history

**DOI:** 10.1002/joa3.12829

**Published:** 2023-02-20

**Authors:** Jun‐ichi Noiri, Hiroki Konishi, Hiroki Matsuzoe

**Affiliations:** ^1^ Department of Cardiovascular Medicine Yodogawa Christian Hospital Osaka Japan

**Keywords:** atrial fibrillation, atrial septal defect, pulmonary vein isolation, surgical patch closure, transthoracic echocardiography

## Abstract

Limited studies report on the status of surgical closure patches for atrial septal defect (ASD) in the remote period. In our case, transthoracic echocardiography revealed a fistula of ASD patch before pulmonary vein isolation for atrial fibrillation. Preoperative Imaging examinations aid in evaluating the effect of the needle puncture around the artificial material of the atrial septum and catheter manipulation for patients with a history of ASD closure.

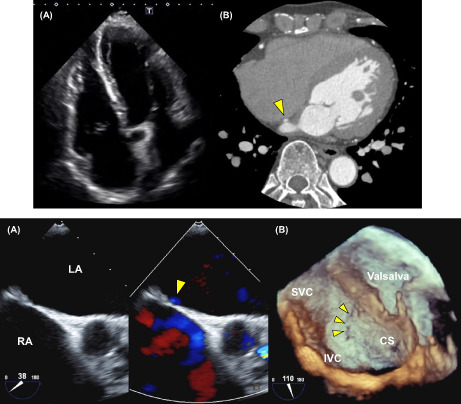

A 72‐year‐old man was referred to our department due to palpitations secondary to paroxysmal atrial fibrillation. He had undergone surgical closure of an atrial septal defect (ASD) approximately 50 years prior. Electrocardiogram revealed paroxysmal atrial fibrillation (AF). Transthoracic echocardiography (TTE) revealed dilation of the left atrium and right ventricle (Figure 1A). The right ventricular dilation was consistent with the history of ASD.

**FIGURE 1:**
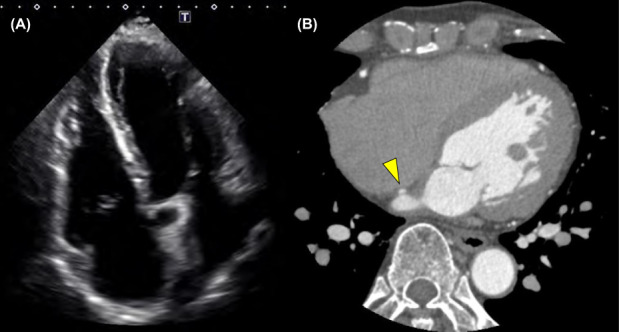
Transthoracic echocardiography image of the dilated left atrium and right ventricle. (A) Dilation of the left atrium and right ventricle. Slightly impaired right ventricular systolic function (left atrial dimension, 41.4 mm; left atrial volume, 58.2 mL; left ventricular ejection fraction, 57.9%; basal right ventricular dimension, 49.4 mm; mid‐right ventricular dimension, 33.5 mm; longitudinal right ventricular dimension, 106.2 mm; and fraction area change of the right ventricle, 30.7%) was also noted. The right ventricle dilation is consistent with the history of an atrial septal defect (ASD). No leakage was identified around the patch. (B) Coronary angiography CT performed 7 years previous revealed contrast leakage from the left to the right atrium, suggestive of a fistula (arrowhead).

According to the diagnosis of AF, edoxaban (30 mg/day, the recommended dose of oral anticoagulant in Japan for patients <60 kg), bisoprolol (0.625 mg/day), and amiodarone (50 mg/day) were prescribed (CHADS_2_ score; 2 points, CHA_2_DS_2_‐VASc score; 3 points). Prior to radiofrequency catheter ablation, preoperative cardiac imaging evaluation of the ASD closure patch was planned.

Although atrial septal shunt flow was not detected using TTE, coronary angiography computed tomography (CT) revealed an atrial shunt (Figure 1B). Transesophageal echocardiography (TEE) identified leakage and risk of transseptal puncture during the ablation procedure. Doppler mode detected flow through the peri‐closure patch of the ASD (Figure 2A and Movie S1). Further, three‐dimensional echography revealed a craniocaudal fistula along the posterior margin of the fossa ovalis, located slightly cephalad from the opening of the coronary sinus (Figure 2B and Movie S2).

**FIGURE 2:**
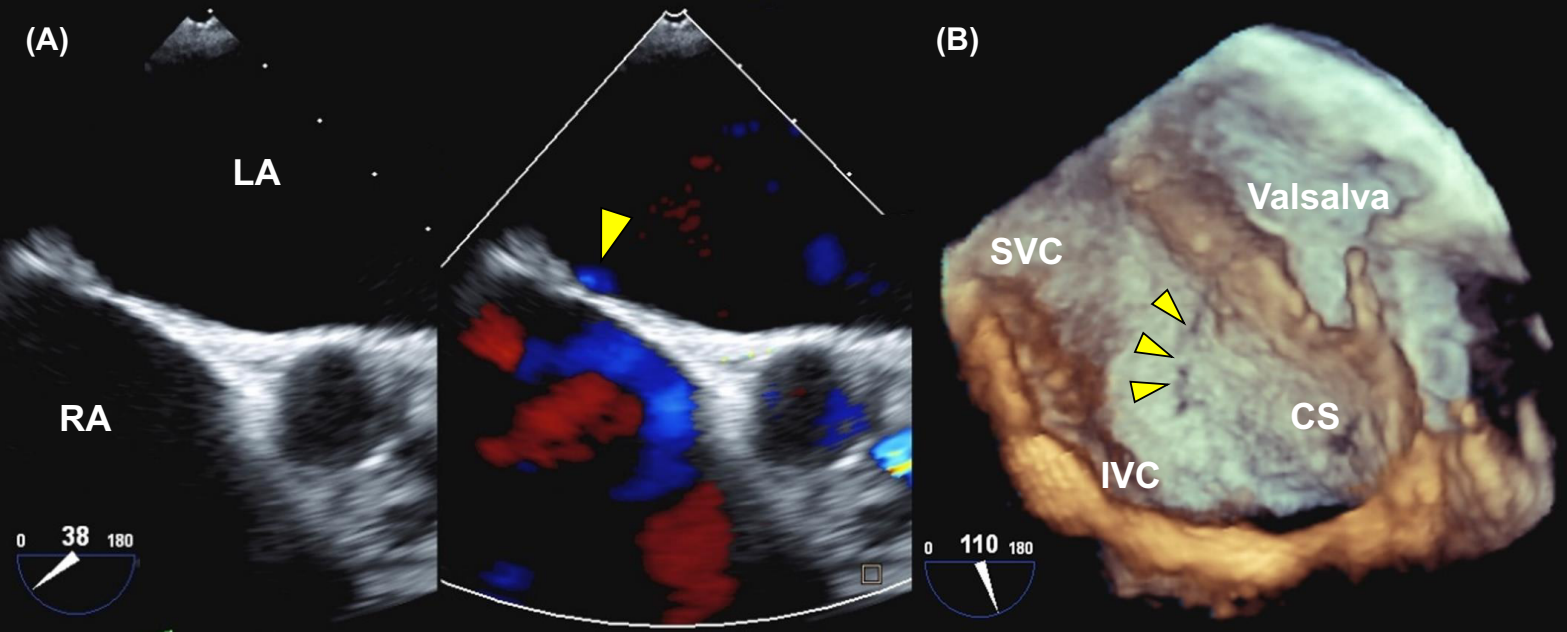
Transesophageal echocardiographic images of peri‐closure patch leakage. (A) Transesophageal echocardiography in Doppler mode detected flow from the left atrium (LA) to the right atrium (RA) through the peri‐closure patch of the ASD, suggestive of a residual shunt. (B) Three‐dimensional echography revealed a craniocaudal fistula (arrowhead) along the posterior margin of the fossa ovalis, located slightly cephalad from the opening of the coronary sinus (CS). IVC, inferior vena cava; SVC, superior vena cava.

Based on the location of the slit identified by TEE prior to the ablation procedure, we aimed a sheath to the slit using the intracardiac echocardiography (ICE) AcuNav catheter (Biosense Webster Inc) and successfully passed a guidewire through to the left atrium without a needle puncture. To avoid increasing the leakage due to manipulation of a large‐diameter sheath, an 8.5‐Fr steerable sheath was only inserted into the left atrium through the slit followed by extensive encircling pulmonary vein isolation (PVI). PVI was performed with a fast anatomical mapping image (Figure S3). Post‐ablation, the pulmonary‐to‐systemic blood flow (Qp/Qs) ratio was measured using the oxygen saturation method. The Qp/Qs ratio was calculated to be 1.0, indicating that our procedure did not induce a significant increase in leakage.

Over 6 months of follow‐up, this patient had no recurrence of AF nor any evidence of increased leakage.

Surgical closure of atrial septal defects (ASDs) has been a standard modality of treatment for decades. Since the first reports of surgical ASD closure in 1948, more than 70 years of experience have resulted in a safe and effective surgery with minimal mortality and complications. However, prolonged follow‐up is rare for patients with a history of surgical treatment for ASD. Thus, to the best of our knowledge, limited studies report the long‐term status of the closure patch.

Those studies that have reported the long‐term outcomes after surgical repair of ASD generally neglect to evaluate the status and material of the closure patch.^1^ A small sample‐size study reported the status of ASD patch 11–26 (median, 22) years after the surgery with a small residual ASD in 2 of 38 patients (5%).^2^ Future research is warranted to elucidate the typical status of the surgical closure patch over time. In our case, since the patient underwent ASD surgical closure at approximately 20 years of age, the influence of mismatch between the closure patch and native foramen caused by growth would be small.

In patients with surgically repaired ASDs, the transseptal puncture method can be performed via the neighboring native atrial septum tissue or the patch itself.^3^ However, as the ASD patch gets older, catheter manipulation is limited and difficult because of the extensive scarring and fibrosis of the patch. Moreover, in the case of a Gore‐Tex patch is difficult to penetrate due to its resistant texture.^3^ We utilized the peri‐patch leak for transeptal access to avoid difficulty in penetration of the ASD patch. Preoperative imaging should be conducted to evaluate the effect of the needle puncture around the artificial material of the atrial septum and catheter manipulation should be considered.^4^ The transseptal approach is increasingly used for the catheterization of valvular diseases and atrial fibrillation. Furthermore, the evolution of a variety of interventions including the transseptal approach necessitates attention to the importance of site‐specific transseptal access,^3^ which emphasizes the importance of preoperative imaging investigations. Herein, we describe the pre‐operative imaging evaluation required for procedures involving transseptal punctures in patients with a history of surgical treatment for ASD. TTE is the most convenient method for ASD screening. Residual ASD can be diagnosed by dilation of the right atrium or right ventricle, abnormal motion of the right ventricular septum, defective foramen, and short‐circuit blood flow. In the present case, TTE did not reveal the residual defect, although the bubble protocol may have detected residual ASD. TEE can be used to assess the morphology of the defect, especially in measuring the maximum diameter and evaluating the margins. TEE also offers the advantage of enabling intraoperative assessment of the morphology of the remaining ASD. Cardiac CT can determine the size of the ASD with the same accuracy as that of TEE and is also useful in identifying abnormalities in the intracardiac structure and measuring systemic and pulmonary blood flows. Additionally, the Qp/Qs ratio obtained by CT measurements, an index for comparison of shunt blood flow before and after the transseptal procedure, is comparable to that obtained by invasive catheterization.^5^ ICE is also practical for intraoperative imaging evaluation, permitting direct visualization of the endocardium and precisely locating the needle and the sheath against the atrial septum. Thus, understanding the characteristics of the imaging modality used is essential for accurate preoperative evaluation of the ASD closure patch. In the present case, preoperative cardiac CT and TEE detected a minor peri‐closure patch leak, providing guidance for PVI.

Here, we reported a case of ASD, detected by preoperative TTE and cardiac CT, where a transseptal approach via minor peri‐closure patch leak was used. Imaging examinations provided important indications informing puncture sites. Evaluation of the status of the closure patch before using a transseptal puncture approach in patients with a history of treatment for ASD has been emphasized, although the follow‐up data of most patients with a history of surgical closure of ASD long time ago are often unavailable because of censored follow‐up. Thus, understanding the utility and benefits of preprocedural multi‐modality imaging to assess the ASD closure patch may improve the utility of the transseptal approach.

## FUNDING INFORMATION

This research did not receive any specific grant from funding agencies in the public, commercial, or not‐for‐profit sectors.

## CONFLICT OF INTEREST STATEMENT

The authors have nothing to disclose.

## ETHICS APPROVAL STATEMENT

Approval was obtained from the local ethics committee.

## PATIENT CONSENT STATEMENT

Written informed consent was obtained from the patient for the publication of this case report.

## PERMISSION TO REPRODUCE MATERIAL FROM OTHER SOURCES

Not applicable.

## CLINICAL TRIAL REGISTRATION

Not applicable.

## INFORMED CONSENT

N/A.

## Supporting information


Figure S3

Movie S1

Movie S2
