# Supplementary figures and images for: The importance of pre‐ablation atrial septal evaluation for a patient with surgical patch closure history
Source: J Arrhythm. 2023 Feb 20;39(2):224–6. doi: 10.1002/joa3.12829 (PMC10068924; doi:10.1002/joa3.12829)

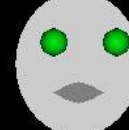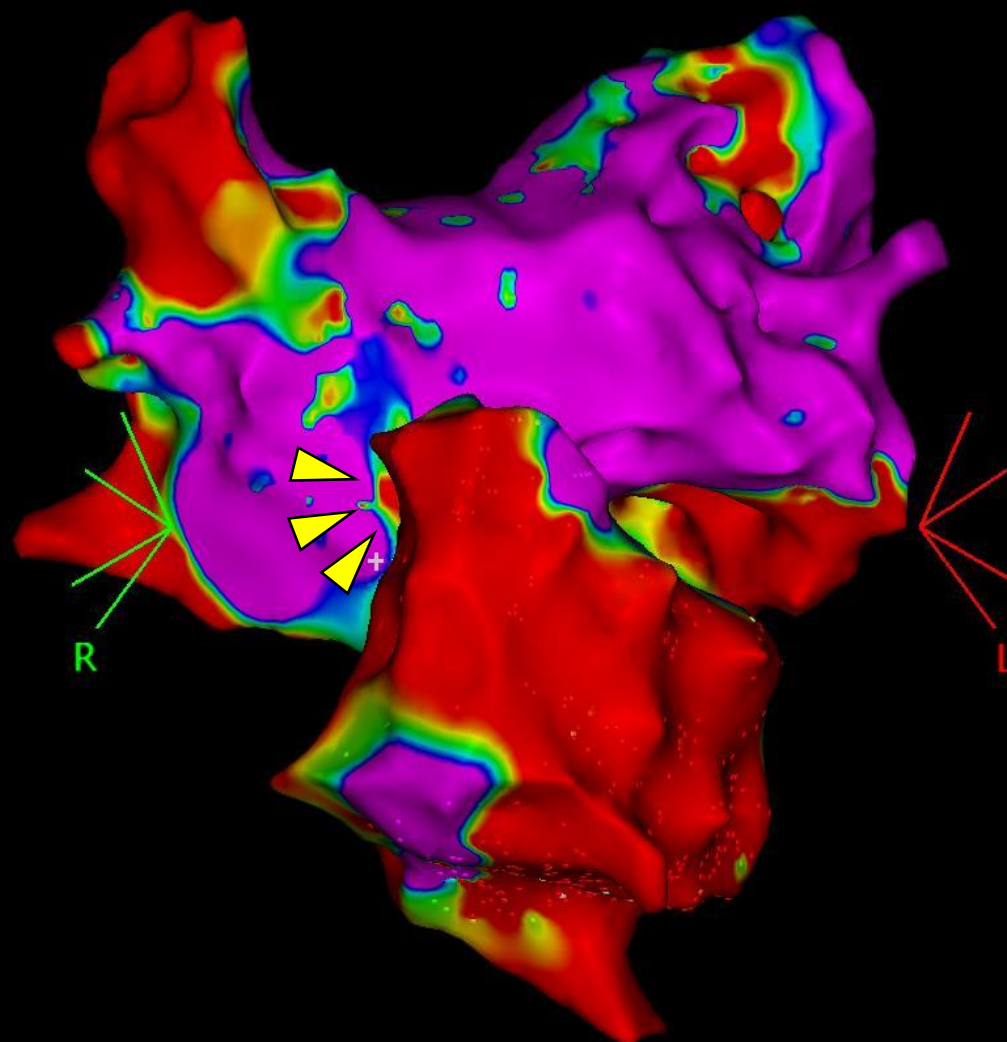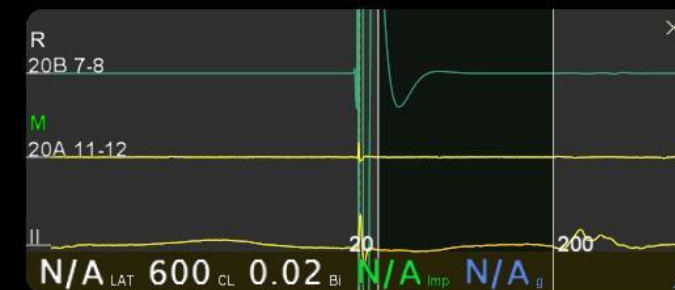

1.61

RAO

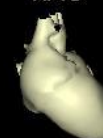

0% ———— + ———— +

AP PA LAO RAO LL RL INF SUP

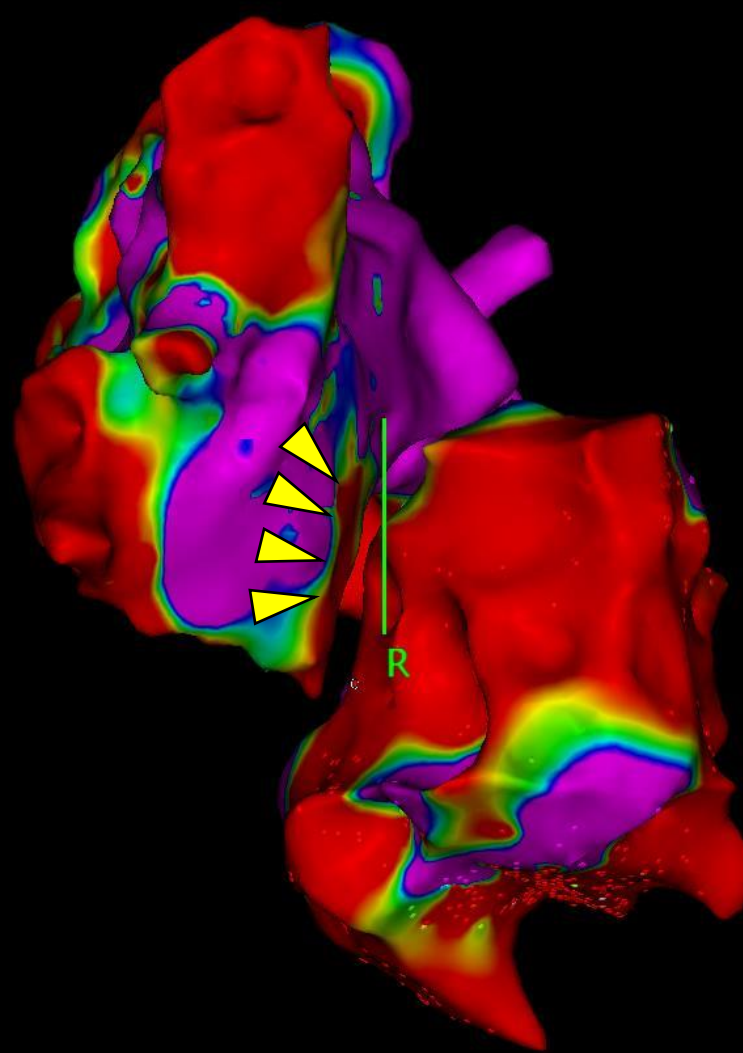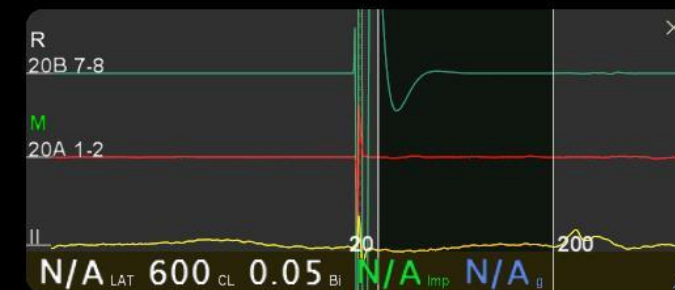

1.61

RL

0%

AP PA LAO RAO LL RL INF SUP

Supplement: Supplementary file 1 — Figure S3 [file JOA3-39-224-s001.pdf]
